# Training Pharmacy Students in Self-Medication Counseling Using an Objective Structured Clinical Examination–Based Approach

**DOI:** 10.1177/23821205211016484

**Published:** 2021-05-31

**Authors:** Imaneh Farahani, Samieh Farahani, Maira Anna Deters, Holger Schwender, Stephanie Laeer

**Affiliations:** 1 Institute of Clinical Pharmacy and Pharmacotherapy, Heinrich Heine University Duesseldorf, Duesseldorf, North Rhine-Westphalia, Germany; 2 Mathematical Institute, Heinrich Heine University Duesseldorf, Duesseldorf, North Rhine-Westphalia, Germany

**Keywords:** Pharmacy education, pharmacy students, OSCE, self-medication, counseling

## Abstract

**Introduction:**

Pharmacists play an important role in ensuring the safe, effective, and rational use of drugs in self-medication. Given the potential risks of self-medication, adequate training on self-medication counseling should be provided to pharmacy students during their academic education. Objective structured clinical examinations (OSCEs) could be used to train pharmacy students in these skills. This study evaluated the efficacy of an OSCE-based approach for training pharmacy students in self-medication counseling and communication skills.

**Methods:**

This randomized controlled study was conducted among pharmacy students using a pre-post design. The intervention group completed OSCE-based self-medication training, while the control group collected counseling-relevant information from summaries of product characteristics of over-the-counter drugs. The counseling and communication skills of both groups before and after training were assessed by completing OSCEs. The participants completed a self-assessment questionnaire on self-confidence and self-perceived proficiency before each OSCE encounter and a satisfaction survey at the end of the seminar.

**Results:**

Students were generally satisfied with the seminar. While the OSCE-trained group demonstrated significantly greater increases in counseling skills and self-confidence and self-perceived proficiency than the control group, both groups had similar increases in communication skills.

**Conclusion:**

The present study suggests that applying OSCEs as a learning tool for self-medication counseling is beneficial for improving students’ counseling skills as well as self-confidence and self-perceived proficiency. These results support the inclusion of OSCEs in pharmacy education and highlight its potential to bridge gaps between knowledge and practice.

## Introduction

Self-medication, defined as “the selection and use of medicines by individuals to treat self-recognized illnesses or symptoms,”^1^ plays an important role in health care by providing patients direct and rapid access to treatment.^2^ It offers patients an active role in their health care, allowing them to personally manage non-critical conditions with non-prescription medicines (NPMs).^2,3^ Access to NPMs, often called over-the-counter (OTC) medications,^3^ varies by country, and, for example, may be available at pharmacies as well as retail stores in some countries.^4,5^ Proper self-medication practices might provide economic benefits, such as reducing the need for medical consultations and the costs of community-funded healthcare programs.^2^ Unfortunately, self-medication comes with potential risks, including, but not limited to, incorrect self-diagnosis or choice of therapy, inadequate administration, inappropriate dosages, excessively prolonged use, dependence, abuse, and contraindications or interactions, which could lead to “an increase in drug-induced disease and wasteful public expenditure.”^2^ Self-medication can also delay the diagnosis and treatment of serious medical conditions or mask the symptoms of a serious condition.^6^ Nevertheless, patients are not always aware of the potential risks of NMPs.^7^ To ensure the safe, appropriate, and effective application of self-medication, pharmacists play an important role.^8^

Pharmacists are experts in drug therapy^9^ and can provide adequate counseling to ensure self-medication is performed appropriately by educating patients about a healthy lifestyle, recommending and advising about NPM-treatments, and referring patients to physicians when symptoms indicate a potentially serious condition.^10^ Ample research supports the beneficial impact of pharmacist intervention in NPM therapy.^8,11,12^ For example, Eickhoff et al^8^ reported that community pharmacists found drug-related problems (DRPs) in 17.6% out of 12 567 self-medication requests (ie, approximately 1 out of 5 encounters), with “inappropriate self-medication, inappropriate requested drug, duration of drug use too long (including abuse), and the wrong dosage” the most frequently reported. In addition, they outlined that according to the pharmacists participating in the study approximately 90% of DRPs could partially or completely be solved, highlighting pharmacists’ important role in detecting DRPs and advising patients on the proper and safe use of medicines during self-medication. Nevertheless, several studies indicate that community pharmacy staff’s (including pharmacists) counseling skills regarding self-medication could be improved.^13-16^ For example, Watson et al^16^ indicate poor consultation performance in community pharmacies mostly due to inadequate information gathering or advice provision. It is vital to gather pertinent details from patients and disclose relevant information to them to address their conditions and therapy appropriately.^1,17^ When counseling patients on NPMs, a pharmacist is responsible for assessing whether a patient can be self-treated within the pharmacists’ scope of practice or a referral to a physician is necessary.^18^

Given the potential risk of self-medication^2,6^ and the room for improvement in pharmacists’ counseling skills,^13-15^ pharmacy students should receive appropriate training during their academic education. One strategic teaching approach could be the incorporation of objective structured clinical examinations (OSCEs) in training pharmacy students. OSCEs have the potential to bridge the gap between academic knowledge and practical application.^18^ OSCEs can be formative or summative, with formative OSCEs functioning as learning tools and summative OSCEs used for evaluating clinical skills or knowledge.^19^ Although, OSCEs are meanwhile widely implemented in pharmacy education, there is a lack of investigations evaluating improvements in NPM counseling skills from formative OSCEs, particularly those with peer-based training, with most studies focusing on the use of OSCEs as an assessment tool.^18,20^

To address this need, this study employed a randomized controlled design to evaluate the effect of an OSCE-based training approach on self-medication counseling skills of pharmacy students, focusing on conditions frequently treated by self-medication: headache, heartburn, and diarrhea.^8,21^ The application of OSCEs to teach self-medication counseling in pharmacy students was based on promising results from a prior study at the institution involving diabetes mellitus counseling, however, without a control group.^22^

## Methods

### Operational definitions

For the purpose of this article, the term “formative OSCEs” describes OSCEs used for training the intervention group (OSCE-based training). For the purpose of this article, the term “summative OSCEs” refers to OSCEs for measuring the participants’ skills at baseline (summative pre-training OSCE) and after training (summative post-training OSCE).

### Study design and participants

This randomized controlled trial with a pre-post design was approved by the responsible ethics committee (Number 2018-246-ProspDEuA). The study was conducted between October 2018 and January 2019 during a clinical pharmacy course at Heinrich Heine University Duesseldorf. The clinical pharmacy course and investigation were conducted in the German language.

Fifty-eight students in the eighth and final semester of their pharmacy studies were invited to participate in the study in October 2018. Students were eligible if they signed voluntarily the informed consent form. It was necessary to limit the sample size to 20 participants per group as the study was conducted as part of a self-medication seminar during the clinical pharmacy course in which the time and staff available were limited. Thus, of the students who signed the informed consent form, 40 students were randomly selected, with 20 randomized into the intervention group and 20 into the control group using the statistical software R.^23^ Non-participating students served as support staff digitizing collected data from self-assessment questionnaires or as timekeepers during summative OSCEs.

### Study procedure

**Figure 1.**
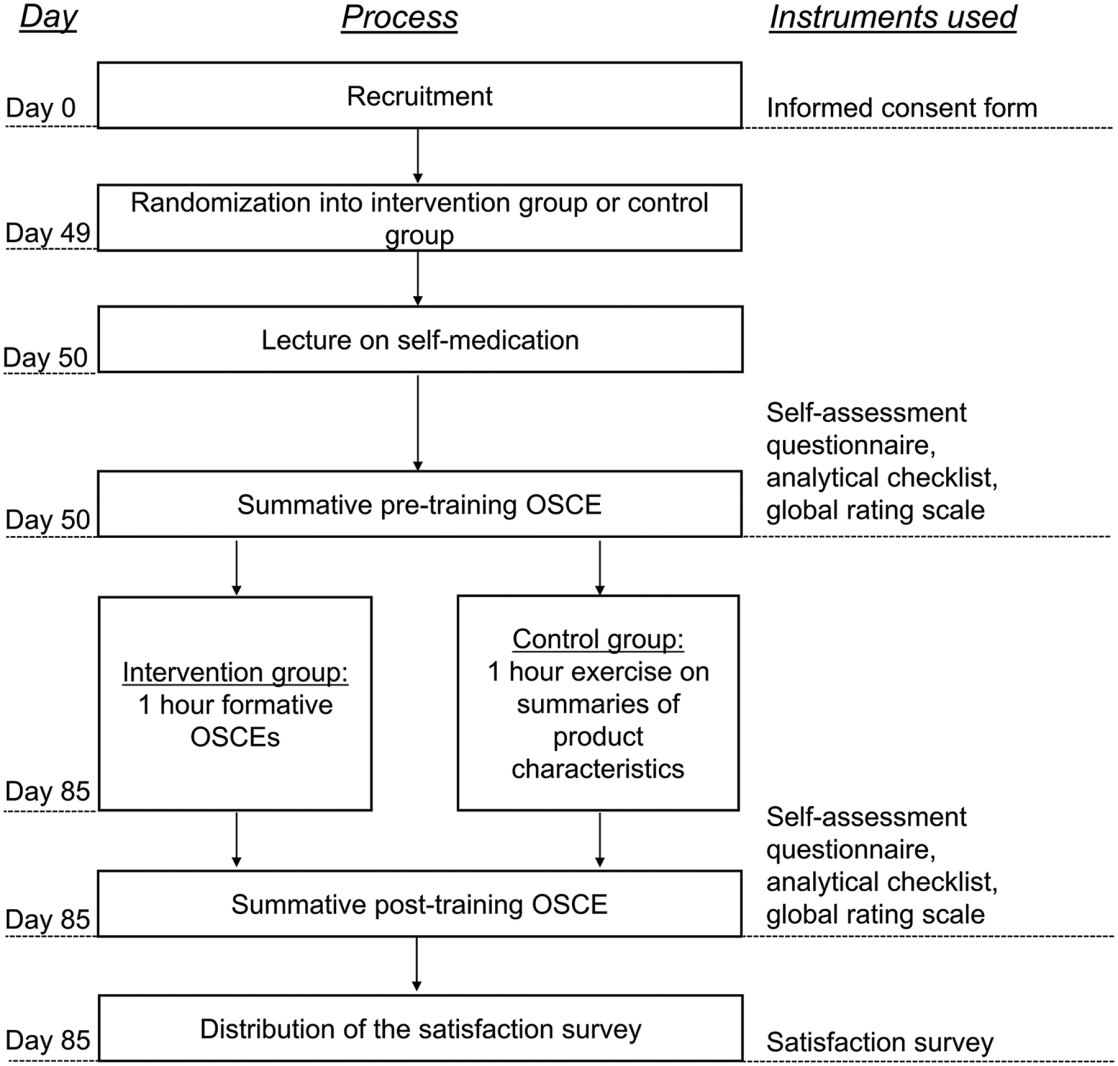
Overview of the study procedure. Abbreviation: OSCE, objective structured clinical examination.

The study (Figure 1) began with recruitment, during which students were informed about and invited to the study. After collecting the informed consent forms, the lots were drawn for determining 40 participants who were randomized into the intervention group or control group. All the students listened to a lecture on self-medication, covering definitions, relevance, legal basis, and clinical aspects focused on headache, heartburn, and diarrhea, to establish comparable basic knowledge. For each indication, the following aspects were addressed:

- an overview of the limits of self-medication
- examples of medicines used for self-medication, for which contraindication, interactions, adverse drug reactions, and a table with (1) information on dosage, (2) duration of intake, and (3) additional important information about the respective medicine (eg, in the case of headache: “prolonged use of any type of pain reliever for headaches can make them worse”) based on the Laven^24^ counseling trio were stated
- additional recommendations

On the same day of the lecture, the participants completed a summative pre-training OSCE evaluating their baseline counseling performance. Five weeks after the summative pre-training OSCE, the participants underwent their assigned training. The intervention group completed formative OSCEs on self-medication, while the control group collected counseling-relevant information from summaries of product characteristics (SmPCs) of OTC drugs (see section *Training* for further details). Immediately following training, participants completed the summative post-training OSCE evaluating the change in their counseling and communication skills (see section *Summative OSCEs* for further details on pre- and post-training OSCEs). Finally, the participants completed an anonymous satisfaction survey.

### Summative OSCEs

The participants completed summative OSCEs before (summative pre-training OSCEs) and after (summative post-training OSCEs) training. Summative pre-training OSCEs assessed the participants’ baseline skills, while summative post-training OSCEs evaluated changes in their OSCE performance after the respective training. Participants filled out a self-assessment questionnaire before each summative OSCE encounter. A pharmacist with experience in community pharmacy developed 20 cases focused on self-medication for headache, heartburn, or diarrhea, 10 each for the summative pre- and post-training OSCEs. All cases were reviewed by another pharmacist. A simulated patient, 1 observer, and 1 participant attended each OSCE encounter. Each OSCE comprised a 1-minute pre-encounter phase, during which the participant had the possibility to read the instruction and the SmPCs, and a 7-minute patient-encounter phase during which the participant assumed the role of the pharmacist and had the task to counsel the simulated patient. The simulated patient initiated each case by directly requesting a product from the participant. The observer evaluated the participant’s performance using a case-specific analytical checklist and global rating scale. To reduce the risk of inter-observer variability due to 2 different observers, the same observer was allocated to each participant for both the summative pre- and post-training OSCEs. Participants received individual feedback from their respective observer immediately after the summative post-training OSCE. The simulated patients and observers were portrayed by 4 faculty members (pharmacists) who were instructed specifically on their tasks. An additional faculty member (pharmacist) coordinated the pre- and post-training OSCEs. OSCEs were completed by 2 participants simultaneously in a single lecture hall.

### Training

The intervention group was divided into 5 groups, each of which trained for 1 hour on 2 summative pre-training OSCE cases concerning the indication completed in the pre-training OSCEs. One case focused on counseling about a drug new to the patient (initiation) and the other about a drug known to the patient (implementation). Each group was provided for the 2 respective cases the following material: the actor description (including patient characteristics), respective SmPCs, the case-specific analytical checklist, and global rating scale. Within these groups, each study participant was instructed to portray the pharmacist. In each group, non-participating students played the role of the simulated patient and/or observer, providing feedback using the case-specific analytical checklist and global rating scale. The intention of involving the non-participating students as simulated patients and/or observers in the formative OSCEs was to let them experience OSCEs as well since the study participants of both groups at least experienced summative OSCEs. Moreover, the participating students had the chance to listen focused to each other’s counseling and provide feedback without simultaneously performing the role of simulated patient. Two instructors were present during training and moved from group to group to answer questions and give feedback.

The control group trained for 1 hour on handling SmPCs for OTC drugs indicated for the treatment of conditions not covered in the OSCEs (obstipation, athlete’s foot, cough, and sore throat). Different indications were used for the control group’s training than in the intervention group’s training because those handled by the intervention group during their training and by both groups in the pre-training OSCEs were already presented in the lecture to both groups and thus, had already been discussed. Participants were required to process the information in the SmPCs in a structured approach by collecting information on each drug, including active ingredients, contraindications, patient situations requiring prior consultation or monitoring by a physician, examples of interactions and adverse drug reactions, dosage and maximal duration of application in the scope of self-medication, important administering information, approved age groups, and examples of additional recommendations the pharmacist could provide for the assigned condition. The content-related aspects to be considered in self-medication counseling regarding the tested indications were already presented to both groups prior to the pre-training OSCEs in the above-mentioned lecture. The control group’s activity on handling SmPCs intended first to facilitate students’ ability to filter out autonomously relevant information on OTC-drugs from the SmPCs as a preparation for the summative post-training OSCEs in which the SmPCs were provided as supporting materials. Second, it purposed to raise the awareness for important elements of self-medication counseling such as contraindications or dosage which need to be considered during counseling by the pharmacist.

### Instruments

#### Analytical checklist

A global analytical checklist modified from previous studies^22,25^ was used to assess the participants’ counseling skills. The modifications were based on the federal pharmacy chamber’s national guidelines for self-medication^26-28^ to account for self-medication counseling requirements and were adapted on a case-specific basis (an example of a case-specific checklist applied for an “initiation” case is depicted in Supplemental Material 1), such that the maximum achievable scores in case-specific checklists varied. The analytical checklist encompassed the sections “greeting,” “medical history,” “drug information” (initiation or implementation), “additional recommendations,” “risk communication,” “goal setting,” “patient involvement,” and where necessary, “additional questions that are necessary in the specific case.” Each section was comprised of 1 or more items. For every correctly performed item in the case-specific analytical checklist 1 point was awarded, if the item was not performed correctly, zero points were awarded.

#### Global rating scale

A global rating scale modified from literature^25^ and previously applied in another study^22^ was used to evaluate participants’ communication skills employing a 6-point Likert scale ranging from 0 (poor behavior) to 5 (optimal behavior). The global rating scale comprised 3 items covering “verbal communication skills,” “non-verbal communication skills,” and “patient-centered communication.”^22^ Both the analytical checklist and global rating scale were completed by the observers during the summative OSCEs.

#### Self-assessment questionnaire

Each participant filled out a self-assessment questionnaire immediately preceding the summative pre- and post-training OSCEs. The questionnaire comprised 7 items intending to rate students’ self-confidence and self-perceived proficiency using a 6-point Likert scale ranging from 0 (“very bad”) to 5 (“very good”) and was based on a self-assessment questionnaire used in studies prior^22,25^ (Supplemental Material 2). The questionnaire for the post-training OSCE also surveyed demographic characteristics, including age, gender, additional education as a pharmaceutical technical assistant, and working in a community pharmacy, counseling patients.

#### Satisfaction survey

Upon completion of the seminar, the participants completed a satisfaction survey comprising 8 items rated on a 6-point Likert scale from “strongly disagree” to “strongly agree” and 2 open-ended questions (free-text items) concerning what they particularly liked about the seminar and what they would suggest changing. For analysis, the comments on the free-text items were categorized into topics.

### Data analyses and statistical methods

This study analyzed the effects of OSCE training on the analytical checklist, global rating scale, and self-assessment questionnaire scores and surveyed students’ satisfaction with this training method. Point-based scores were converted into percentages or percentage points (p.p.) to enable comparison between the different OSCE cases. A 2-sided Mann-Whitney test was applied for a baseline comparison of the scores between the 2 groups. A 1-sided Wilcoxon signed-rank test applied to the differences between pre- and post-training scores was used to evaluate whether the respective scores increased significantly from pre- to post-training. A 1-sided Mann-Whitney test was used to determine whether score increases from pre- to post-training in the respective scores were significantly greater in the intervention group as compared to the control group. In all statistical tests, the significance level was considered to be alpha = 0.05. Asymptotic *P*-values are stated which were not adjusted for multiple testing. All data were collected in pseudonymous form, except the anonymous satisfaction survey. After analysis, all data were rendered anonymous. The statistical software R^23^ was used for randomization, Microsoft Excel 2019^29^ was used for data entry, and Microsoft Excel 2019^29^ and OriginPro 2019^30^ were used for data analyses.

## Results

### Participants

Of the 58 students in the semester, 46 signed the informed consent form and 40 of them were randomly selected for the study. All the 40 participants attended the summative pre-training OSCE. Participants who did not attend the summative pre-training OSCE, and/or training and/or summative post-training OSCE were excluded from the analyses. Additionally, 1 participant was excluded due to non-standardized conditions during the summative post-training OSCE but could not be excluded from the satisfaction survey due to its anonymous character. Finally, 16 participants in the intervention group and 14 in the control group were included in the analyses of OSCE performance and the self-assessment questionnaire. The demographic characteristics of the participants are depicted in Table 1.

**Table 1.** Demographic characteristics.

	Intervention group	Control group
Age in years	n = 16	n = 12*
Mean (SD)	25.75 (2.84)	24.08 (1.73)
Median (IQR)	25 (4.5)	24 (3)
Range	22-32	22-27
Gender	n = 16	n = 14
Female, n (%)	13 (81.25)	10 (71.43)
Male, n (%)	3 (18.75)	4 (28.57)
Additional education as a pharmaceutical technician assistant	n = 16	n = 14
Yes, n (%)	4 (25)	5 (35.71)
No, n (%)	12 (75)	9 (64.29)
Working in a community pharmacy (counseling patients)	n = 16	n = 13**
Yes, n (%)	3 (18.75)	3 (23.08)
No, n (%)	13 (81.25)	10 (76.92)

Abbreviations: IQR, interquartile range; SD, standard deviation.

* Two participants did not provide information about their age.

** One participant did not provide information about his/her work in a community pharmacy.

### Analytical checklist

**Figure 2.**
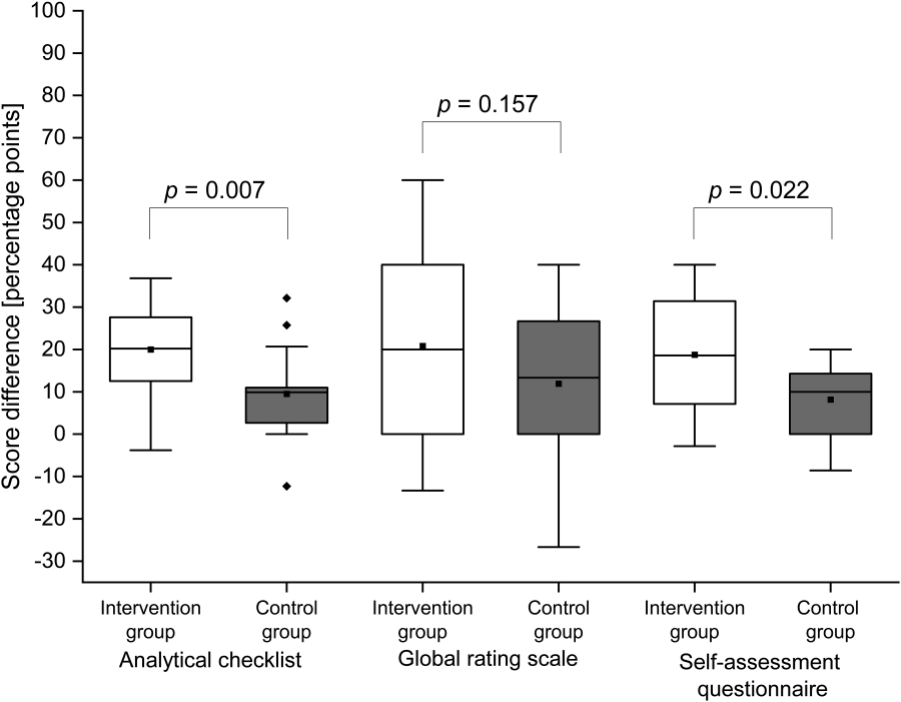
Score differences in percentage points (p.p.). White boxes = intervention group; gray boxes = control group; black squares (■) = mean; horizontal lines = median; black diamonds (♦) = outliers. A 1-sided Mann-Whitney test with a significance level of alpha = 0.05 was used. N = 16 (intervention group) and n = 14 (control group).

The analytical checklist score reflects the participants’ counseling skills, particularly regarding content and structure (Table 2). At baseline, there was no significant difference in the analytical checklist scores between the 2 groups (*P* = .884). Following the respective training, significantly higher scores were observed for both groups in the summative post-training OSCE as compared to the pre-training OSCE (intervention group: *P* < .001; control group: *P* = .007). The intervention group showed significantly greater improvement than the control group (*P* = .007) (Figure 2).

**Table 2.** Intervention and control group scores from the analytical checklist, global rating scale, and self-assessment questionnaire.

Group	Summative pre-training score in %		Summative post-training score in %		Score difference in percentage points		*P*-value*
	Mean (SD)	Median (IQR)	Mean (SD)	Median (IQR)	Mean (SD)	Median (IQR)	
Analytical checklist							
Intervention group	33.47 (7.00)	32.74 (8.47)	53.46 (7.49)	50 (10.73)	19.98 (10.93)	20.19 (15.10)	*P* = .007
Control group	34.16 (10.95)	33.91 (16.93)	43.66 (11.36)	44.51 (21.43)	9.51 (11.16)	9.88 (8.34)	
Global rating scale							
Intervention group	58.33 (14.50)	56.67 (26.67)	79.17 (14.58)	80 (23.33)	20.83 (23.08)	20 (40)	*P* = .157
Control group	63.33 (14.02)	66.67 (6.67)	75.24 (9.58)	73.33 (13.33)	11.90 (17.77)	13.33 (26.67)	
Self-assessment questionnaire							
Intervention group	49.11 (20.05)	47.14 (34.29)	67.86 (13.98)	68.57 (20)	18.75 (14.00)	18.57 (24.29)	*P* = .022
Control group	49.18 (19.51)	50 (40)	57.35 (15.47)	55.71 (22.86)	8.16 (9.43)	10 (14.29)	

N = 16 (intervention group) and n = 14 (control group).

* A 1-sided Mann-Whitney test with a significance level of alpha = 0.05 was applied to the score difference (from pre- to post-training) of both groups in the respective scores.

Abbreviations: IQR, interquartile range; SD, standard deviation.

### Global rating scale

The global rating scale score represents the participants’ communication skills (Table 2). At baseline, there was no significant difference in the global rating scale scores between the 2 groups (*P* = .342). These scores significantly increased for both the intervention group (*P* = .002) and the control group (*P* = .015) in the summative post-training OSCE as compared to the pre-training OSCE. The intervention group tended to have a greater score increase in communication skills (intervention group: mean change = 20.83 p.p. (SD = 23.08 p.p.) and median = 20 p.p. (IQR = 40 p.p.); control group: mean change = 11.90 p.p. (SD = 17.77 p.p.) and median = 13.33 p.p. (IQR = 26.67 p.p.)), although the increase was not significantly greater in the intervention group as compared to the control group (*P* = .157) (Figure 2).

### Self-assessment questionnaire

The self-assessment questionnaire score reflects the participants’ self-confidence and self-perceived proficiency (Table 2). At baseline, there was no significant difference in the self-assessment questionnaire scores between the 2 groups (*P* = 0.787). While both groups showed a significant increase in the self-assessment questionnaire scores from the summative pre- to post-training OSCEs (intervention group: *P* < .001; control group: *P* = .007), this was significantly higher for the intervention group as compared to the control group (*P* = .022) (Figure 2).

### Satisfaction survey

A total of 22, who attended both summative OSCEs and the respective training, completed the satisfaction survey, which did not distinguish between the 2 groups (Tables 3 and 4). The majority of participants approved of the OSCE seminar, with 72.73% agreeing (slightly agree, agree, and strongly agree summarized) that OSCEs should be implemented in the clinical pharmacy course.

**Table 3.** Results of the satisfaction survey.

	Proportions of responses in %					
	Strongly disagree	Disagree	Slightly disagree	Slightly agree	Agree	Strongly agree
I enjoyed the OSCE seminar.	4.55	13.64	4.55	27.27	45.45	4.55
During the OSCEs, I was able to determine my strengths and weaknesses.	0	4.55	31.82	22.73	31.82	9.09
The OSCE seminar conveyed safety in dealing with patients in the community pharmacy.	4.55	18.18	22.73	22.73	18.18	13.64
The OSCE cases were practice-oriented.	0	4.55	18.18	31.82	40.91	4.55
The OSCE cases were too easy.	0	9.09	27.27	27.27	22.73	13.64
The OSCE cases were too difficult.	36.36	31.82	27.27	4.55	0	0
Two days for the OSCE seminar were sufficient.	4.55	13.64	27.27	18.18	13.64	22.73
OSCEs should be implemented in future clinical pharmacy course to train counseling skills.	4.55	9.09	13.64	13.64	40.91	18.18

N = 22.

Abbreviation: OSCE, objective structured clinical examination

**Table 4.** Example topics of comments from free-text items of the satisfaction survey.

Free-text item	Topic of comments*
I particularly liked the following at the OSCE seminar:	• Patient counseling
	• Receiving feedback after summative post-training OSCE
	• Friendly faculty members
I would change the following:	• Long waiting times for the summative OSCEs
	• Training on the summary of product characteristics was unnecessary (control group’s training)
	• OSCE cases were too easy

* The 3 most frequent topics of comments per item are shown.

Abbreviation: OSCE, objective structured clinical examination

## Discussion

This randomized controlled study showed that our OSCE-based training approach was well accepted by pharmacy students and provides an effective approach for teaching self-medication counseling. OSCE-based training improved students’ self-confidence and self-perceived proficiency, as well as their counseling skills, compared to a non-OSCE-trained control group. However, OSCE-based training did not result in a significantly greater increase of communication skills in the intervention group as compared to the control group.

Our findings support the use of OSCEs as a method for training self-medication counseling skills to pharmacy students, with our OSCE-based training resulting in significantly greater improvements in counseling performance in the intervention group compared to the control group. However, there is still controversy regarding the efficacy of formative OSCES in the literature.^19,31^ Moreover, few investigations focus on the use of NPM-related OSCEs. For example, Hastings et al^18^ investigated the effect of summative NPM OSCEs on students’ final grades. They refined the NPM elective course for pharmacy students by including case-based small group periods, which incorporated role-playing (similar to the formative OSCEs in our study) and other tasks, and added a final summative OSCE. They found similar overall grades compared to previous years where OSCEs were not part of the overall grade. However, they did not report further results regarding the efficacy of their refined elective course on their summative OSCEs. Our research evaluated the efficacy of a peer interaction–based OSCE training approach in a randomized controlled design and found a greater improvement in the summative post-training OSCE for the OSCE-trained intervention group compared to the control group, although there is still room for improvement (mean post-training score in the analytical checklist: 53.46% (SD = 7.49%), median post-training score: 50% (IQR = 10.73%) for the intervention group). In contrast to that, Hastings et al^18^ reported an average grade of 78% in the 3-case OSCE final, where students completed 2-hour credit courses for 2 semesters. We hypothesize that longer or more frequent training will lead to higher OSCE scores.

The use of formative OSCEs in this study did not lead to a significantly greater improvement of the communication skills in the intervention group as compared to the control group, although both groups displayed significant improvement from the summative pre- to post-training OSCEs. It might be possible that longer and more frequent OSCE training sessions would result in a significantly higher increase in the intervention group’s global rating scale score as compared to the control group. This assumption is also indicated by findings in the literature.^18,32^ For example, a randomized controlled study by Cannick et al^32^ investigating a brief 2-hour communication skills training for dental students assessed by OSCEs found no significant differences from baseline to post-test between the intervention and control group. They concluded that the brief training was insufficient and that comprehensive training with frequent reinforcements might be more beneficial. However, it should be considered that in the study at hand, the final scores of the global rating scale (post-training scores) show only little room for further improvement in both groups.

Increases in self-assessment questionnaire scores reflect increases in participants’ self-confidence and self-perceived proficiency. This study found significant increases in self-confidence through the application of OSCEs, in agreement with findings in the literature.^22,33-35^ Moreover, the majority of students in this study agreed that OSCEs should be implemented in future clinical pharmacy courses for training their counseling skills. These findings support students’ acceptance of OSCEs, which is in line with findings of other studies.^22,33,36^ Although the control group’s training with the SmPCs was rather disliked by the students, the positive results, including the significant increase in the analytical checklist score, global rating scale score, and self-assessment questionnaire score from pre- to post-training OSCEs, indicate a beneficial contribution on students’ counseling skills. Nevertheless, regarding the analytical checklist and self-assessment questionnaire, the OSCE-trained group was superior.

We assume that using a pre-test/post-test design might have led to underestimating the effect of the intervention (OSCE-based training). The pre-training OSCE might have caused a learning effect as the students might be faced with their weaknesses as previously assumed by other researchers.^37^ As such, it is possible that removing the pre-training OSCE from this study would better reveal the effects of the intervention, including in the participants’ communication skills.

We are aware of some limitations. The analytical checklists and global rating scale were only available to the intervention group during their training to enable the students to provide each other adequate feedback and were collected again after the 1-hour training. The checklists were not provided to the control group. Although we cannot completely exclude a potential impact of the provision of the checklists, we assume that knowledge of the checklists would probably not substantially affect the performance of the intervention group compared to the control group during the summative post-training OSCEs. This assumption is supported by the findings of Cole et al.^38^ In particular, they compared the OSCE scores of students who attended a peer-taught training session to the scores of students who did not attend the session. Both groups were provided with scoring rubrics during the semester. Although differences in student scores for each skill were not statistically significant between both groups, they found a significant difference in the overall OSCE score favoring the group which attended the training session. The rationale of providing the checklists to the intervention group was to set a framework for adequate peer feedback while coping with limited staff available.

The decrease in participation rate at the post-training OSCE, which was the final clinical pharmacy seminar day in the semester, might be due to competing demands in their time at the end of the semester because of pending exams. Moreover, in educational research “contamination” can occur, such as students randomly assigned to different groups share information.^39^ To mitigate this possible bias, the post-training OSCEs were conducted immediately after the training on the same day. Moreover, due to the lack of staff, only 2 OSCE encounters could take place at 1 time. Thus, some students had long waiting times for the OSCEs which was criticized in the satisfaction survey. This might have negatively influenced the results of the satisfaction survey.

Despite these limitations, the results show a valuable benefit of applying an OSCE-based training approach in improving pharmacy students’ self-medication counseling skills. As pharmacists play an important role to ensure the safe, appropriate, and effective application of self-medication,^8^ and literature indicates room for improvement of pharmacists’ self-medication counseling skills,^13-16^ we suggest that an OSCE-based training approach has the potential to contribute to the future pharmacists’ education.

## Conclusion

This study found that our OSCE-based training was widely accepted by pharmacy students and provides an effective method for training self-medication counseling. Applying OSCEs as a learning tool in pharmacy education is beneficial, improving both the students’ counseling skills as well as self-confidence and self-perceived proficiency. These findings support the inclusion of this strategic educational approach throughout pharmacy education and highlight its potential for bridging gaps between knowledge and practice.

## Supplemental Material

sj-pdf-1-mde-10.1177_23821205211016484 – Supplemental material for Training Pharmacy Students in Self-Medication Counseling Using an Objective Structured Clinical Examination–Based Approach Click here for additional data file. Supplemental material, sj-pdf-1-mde-10.1177_23821205211016484 for Training Pharmacy Students in Self-Medication Counseling Using an Objective Structured Clinical Examination–Based Approach by Imaneh Farahani, Samieh Farahani, Maira Anna Deters, Holger Schwender and Stephanie Laeer in Journal of Medical Education and Curricular Development

sj-pdf-2-mde-10.1177_23821205211016484 – Supplemental material for Training Pharmacy Students in Self-Medication Counseling Using an Objective Structured Clinical Examination–Based Approach Click here for additional data file. Supplemental material, sj-pdf-2-mde-10.1177_23821205211016484 for Training Pharmacy Students in Self-Medication Counseling Using an Objective Structured Clinical Examination–Based Approach by Imaneh Farahani, Samieh Farahani, Maira Anna Deters, Holger Schwender and Stephanie Laeer in Journal of Medical Education and Curricular Development
