# Supplementary material for: Training Pharmacy Students in Self-Medication Counseling Using an Objective Structured Clinical Examination–Based Approach
Source: J Med Educ Curric Dev. 2021 May 31;8:23821205211016484. doi: 10.1177/23821205211016484 (PMC8170271; doi:10.1177/23821205211016484)
Supplement: sj-pdf-1-mde-10.1177_23821205211016484 – Supplemental material for Training Pharmacy Students in Self-Medication Counseling Using an Objective Structured Clinical Examination–Based Approach [file sj-pdf-1-mde-10.1177_23821205211016484.pdf]

Ibuprofen 400 mg – Initiation

Participant code: ______________________________

Date: ____

Observer: ____________________________________

Please comment if statements are made that you are not sure whether they are correct or whether they can be scored.

(The standardized patient asks for ibuprofen 400 mg.)

Section 1: Greeting: The pharmacist …	Addressed	Comment
**1.1 Introduces himself/herself**<br>*Pharmacist: Hello, my name is ...*		
**1.2 Identifies the patient**<br>*Pharmacist: Who is the medicine for?*<br>*Patient: For me.*		
**1.3 Checks/adds to the patient's record**<br>*Pharmacist: Are you saved in our customer file?*<br>*Patient: No.*		
**Section 2: Medical history: The pharmacist …**	**Addressed**	**Comment**
**2.1 Asks which prescription drugs the patient is taking**<br>*Pharmacist: What prescription drugs do you take?*<br>*Patient: I am not taking any prescription drugs.*		
**2.2 Asks which self-medication products including phytotherapeutics, etc. the patient is taking**<br>*Pharmacist: What non-prescription drugs are you taking? For example, over-the-counter drugs, herbal drugs, drugs from the drugstore, or dietary supplements.*<br>*Patient: Nothing.*		
**2.3 Asks about other medical conditions (i.e. chronic diseases, allergies, pregnancy/lactation period, kidney/liver diseases)**<br>*Pharmacist: Is there anything else I should know about your health state in order to be able to advise you optimally?*<br>*Patient: What do you mean by that?*<br>*Pharmacist: For example, other chronic diseases, allergies, pregnancy, breastfeeding?*<br>*Patient: No, there is nothing.*		
**2.4 Asks about present symptoms (checks patient's self-diagnosis)**<br>*Pharmacist: What exactly are the complaints?*<br>*Patient: I have a headache with dull-pressing pain on both sides.*		
**2.5 Asks how long the symptoms have been present**<br>*Pharmacist: How long do you have the symptoms?*<br>*Patient: Since this morning. I think I didn't get enough sleep last night.*		
**2.6 Asks how often the symptoms appear**<br>*Pharmacist: How often does it happen that you have a headache?*<br>*Patient: Very rarely. Maximal once a month.*		
**2.7 Asks when the symptoms appear (to find out potential reasons for headache)**<br>*Pharmacist: And when do you always get a headache?*<br>*Patient: Mostly if I didn't sleep enough, like today. Or if I drink too much alcohol, but this happens very rarely.*		
**2.8 Asks about accompanying symptoms**<br>*Pharmacist: What other complaints/symptoms do you have?*<br>*Patient: Nothing else.*		
**2.9 Asks whether the symptoms have already been clarified by a physician**<br>*Pharmacist: Have the complaints been checked out by the doctor before?*<br>*Patient: I consulted the doctor a while ago. He said it was only a tension headache.*		

**2.10 Asks if something has been done about the symptoms**<br>*Pharmacist: Have you already taken something for your headache today?*<br>*Patient: No, nothing at all.*			
**2.11 Checks whether it is a first-time application**<br>*Pharmacist: Have you ever taken ibuprofen tablets?*<br>*Patient: No, not yet. A colleague recommended the tablets to me.*			
**Section 3.1 - Initiation of therapy: The pharmacist ...**	**Addressed**	**Correct**	**Comment**
**3.1.1 Provides information about the effects and benefits of the drug**<br>*Pharmacist: Ibuprofen is an active ingredient that is very good at relieving pain.*			
**3.1.2 Explains the correct single dose**<br>*Pharmacist: Please take one tablet as required.*			
**3.1.3 Explains the maximum daily dose**<br>*Pharmacist: Do not take more than 3 tablets a day.*			
**3.1.4 Give the patient information regarding the dosing interval**<br>*Pharmacist: Keep an interval of at least 6 hours between the doses.*			
**3.1.5 Provides specific information on drug application (inhalation, spray, injection; taking with or without food)**<br>*Pharmacist: Please take the tablet with or after food, with a glass of water.*			
**3.1.11 Explains potentially relevant side effects**<br>*Pharmacist: Ibuprofen may cause gastrointestinal problems.*			
**3.1.13 Explains what the patient should do in the event of an adverse drug reaction**<br>*Pharmacist: If these problems occur stop taking ibuprofen immediately and consult your doctor.*			
**3.1.15 Gives additional relevant information/warnings**<br>*Pharmacist: In general, do not take pain relievers on a regular basis.*			
**Section 4 - Supportive measures: The pharmacist ...**	**Addressed**	**Correct**	**Comment**
**4.2 Additional recommendations/supportive measures**<br>*Pharmacist: Avoid known triggers (e.g. alcohol, nicotine). Make sure you have enough rest, get enough sleep, and drink enough liquids. Another recommendation is doing muscle relaxation exercises.*			
**Section 5 - Risk communication: The pharmacist ...**	**Addressed**		**Comment**
**5.2 Decides that self-medication is possible as the limits of self-medication are not exceeded**			
**5.4 Decides to dispense the drug as it is suitable**			
**Section 5 - Risk communication: The pharmacist ...**	**Addressed**	**Correct**	**Comment**
**5.6 Informs the patient when to contact the physician if symptoms persist**<br>*Pharmacist: If your condition does not improve within 3 to 4 days, please see your doctor.*<br>*Patient: Ok.*			
**Section 6 - Goal Setting: The pharmacist ...**	**Addressed**		**Comment**
**6.1 Sets individual goals**<br>*Pharmacist: Can you summarize again how you will proceed now?*<br>*Patient: If required I take one tablet. If I need another tablet I have to wait at least 6 hours before I take another tablet. But I must not take more than 3 tablets a day. And if the headache doesn't get better after 3 to 4 days, I will consult the doctor.*			
**Section 7 - Patient involvement: The pharmacist ...**	**Addressed**		**Comment**
**7.1 Asks the patient for any open questions**<br>*Pharmacist: Do you have any open questions?*<br>*Patient: No, not at the moment.*			
**7.3 Informs the patient that he/ she should contact the pharmacist or physician if they have any questions**<br>*Pharmacist: If you have any questions in the meantime, please do not hesitate to contact your doctor or us.*<br>*Patient: Thank you*			
**Sum**			

The checklist is translated from German. The dialogs in italics are only exemplary formulations. The checklists used in the study vary depending on the specific case and whether it is an "initiation" or "implementation" case.
