# Supplementary material for: Training Pharmacy Students in Self-Medication Counseling Using an Objective Structured Clinical Examination–Based Approach
Source: J Med Educ Curric Dev. 2021 May 31;8:23821205211016484. doi: 10.1177/23821205211016484 (PMC8170271; doi:10.1177/23821205211016484)
Supplement: sj-pdf-2-mde-10.1177_23821205211016484 – Supplemental material for Training Pharmacy Students in Self-Medication Counseling Using an Objective Structured Clinical Examination–Based Approach [file sj-pdf-2-mde-10.1177_23821205211016484.pdf]

How do you rate your counseling competences in the following areas?

1.) How do you rate your competence in self-medication counseling?	very bad	☐	☐	☐	☐	☐	☐	very good
2.) How do you rate your competence in motivating a patient to carry out the therapy?	very bad	☐	☐	☐	☐	☐	☐	very good
3.) How do you rate your competence in actively listening?	very bad	☐	☐	☐	☐	☐	☐	very good
4.) How do you rate your competence to lead through questions?	very bad	☐	☐	☐	☐	☐	☐	very good
5.) How do you rate your competence in structuring a consultation according to the time available?	very bad	☐	☐	☐	☐	☐	☐	very good
6.) How do you rate your competence in providing correct, relevant and useful information to the patient during the consultation?	very bad	☐	☐	☐	☐	☐	☐	very good
7.) How do you rate your competence in transferring specialist knowledge in lay language?	very bad	☐	☐	☐	☐	☐	☐	very good
